# Identification of Two Auxin-Regulated Potassium Transporters Involved in Seed Maturation

**DOI:** 10.3390/ijms19072132

**Published:** 2018-07-22

**Authors:** Rubén Tenorio-Berrío, Marta-Marina Pérez-Alonso, Jesús Vicente-Carbajosa, Leticia Martín-Torres, Ingo Dreyer, Stephan Pollmann

**Affiliations:** 1 Centro de Biotecnología y Genómica de Plantas, Instituto Nacional de Investigación y Tecnología Agraria y Alimentación (INIA), Universidad Politécnica de Madrid (UPM), 28223 Pozuelo de Alarcón, Spain; ruben.tenorio@alumnos.upm.es (R.T.-B.); martamarina.perez@upm.es (M.-M.P.-A.); jesus.vicente@upm.es (J.V.-C.); letimartintorres@hotmail.com (L.M.-T.); idreyer@utalca.cl (I.D.); 2 Centro de Bioinformática y Simulación Molecular (CBSM), Universidad de Talca, 2 Norte 685, 3460000 Talca, Chile

**Keywords:** *Arabidopsis thaliana*, seed size, seed maturation, auxin, indole-3-acetic acid, indole-3-acetamide

## Abstract

The seed is the most important plant reproductive unit responsible for the evolutionary success of flowering plants. Aside from its essential function in the sexual reproduction of plants, the seed also represents the most economically important agricultural product worldwide, providing energy, nutrients, and raw materials for human nutrition, livestock feed, and countless manufactured goods. Hence, improvements in seed quality or size are highly valuable, due to their economic potential in agriculture. Recently, the importance of indolic compounds in regulating these traits has been reported for *Arabidopsis thaliana*. The transcriptional and physiological mechanisms involved, however, remain largely undisclosed. Potassium transporters have been suggested as possible mediators of embryo cell size, controlling turgor pressure during seed maturation. In addition, it has been demonstrated that the expression of K^+^ transporters is effectively regulated by auxin. Here, we provide evidence for the identification of two *Arabidopsis* K^+^ transporters, *HAK/KT12* (At1g60160) and *KUP4* (At4g23640), that are likely to be implicated in determining seed size during seed maturation and, at the same time, show a differential regulation by indole-3-acetic acid and indole-3-acetamide.

## 1. Introduction

Seed size is an essential indicator of plant fitness and a major determinant for agricultural yield. Hence, it is one of the most important traits analyzed for agronomic purposes. Seed formation is divided into two major phases: embryo and endosperm development (or morphogenesis), and seed maturation [1,2]. Maturation is the phase between embryogenesis and the subsequent desiccation phase, marking the period in which nutrients are stored and seed cells expand. During maturation, seeds establish their final size, thus, being a vital phase to be investigated in order to improve not only seed yield, but also seed quality.

During the maturation phase, the seed undergoes important transformations ranging from the accumulation of storage compounds in the endosperm (endospermic seeds) or in cotyledons (non-endospermic seeds), to the acquisition of desiccation tolerance and growth arrest [3,4,5,6]. Seed maturation begins with a transition phase from maternal to filial control [7], followed by a period of embryo growth and filling that is characterized by vast cell growth and expansion, the formation of storage organelles, and the loss of central vacuoles. This latter phase is not only important to ensure proper plant development, but its optimization also represents a crucial target to improve the quality and yield of seed crops.

Several molecular networks controlling seed maturation have been characterized [8]. Firstly, hormonal signaling during maturation is led by abscisic acid (ABA) activity, which is generally considered as the key-hormone driving this developmental process [9,10]. Apart from ABA, other metabolites are also assumed to play crucial roles in the regulation of seed maturation. Among those substances, mono- and disaccharides, and here especially, the relation between sucrose and hexose, play seemingly pivotal roles [7]. In addition, the four transcription factors ABSCISIC ACID INSENSITIVE 3 (ABI3), FUSCA 3 (FUS3), LEAFY COTYLEDON 2 (LEC2), and HEME ASSOCIATED PROTEIN 3 (HAP3) act as master regulators that are known to interact to control different features of seed maturation [11]. All of those regulatory layers of crosstalk generate a very complex network, which is far from being completely elucidated [2].

Auxins are essential plant hormones linked to a wide variety of processes associated with plant growth and development [12,13,14]. Consequently, it is not surprising that auxins have been also reported to substantially affect seed development, determining embryo polarity, and structure [15,16,17,18]. It has been demonstrated that the concentration of the most abundant plant auxin, indole-3-acetic acid (IAA), increases in seeds between eight and 28 days after pollination [19] and that these levels remain high until the seeds undergo germination [20]. Altogether, this strongly suggests an implication of IAA in seed maturation. It has been demonstrated that IAA controls seed size through the action of a number of AUXIN RESPONSE FACTOR (ARF) transcription factors [21,22], which collectively control cell proliferation in maternal integuments. Furthermore, the auxin-mediated regulation of a plasma membrane receptor kinase (FER) involved in regulating cell elongation in seed coats has been proposed [23,24].

Rapid cell expansion during seed maturation generally relies on two principal processes: on the one hand, cell walls have to be softened and, on the other hand, the turgor pressure has to increase. Xyloglucan endotransglycolases/hydrolase (XTH) enzymes are assumed to play an important role in cell wall softening and modification [25,26,27]. Further studies demonstrated that the expression of some *XTH* genes is positively regulated by auxin [28,29]. Rapid cell expansion is achieved through the increase of the cellular turgor pressure, which mainly relies on the controlled import of ions in order to promote the subsequent influx of water. Since potassium is the predominant inorganic ion in plant cells [30], potassium transporters and channels are the most likely candidates in producing osmotic gradients across the plasma membrane and, hence, being the responsible molecular mediators of rapid cell expansion [31]. However, investigation of which channels/transporters are implicated in potassium import during embryo maturation has not yet been pursued. Several K^+^ transporters/channels have been identified in the *Arabidopsis thaliana* genome. They are classified as proton-coupled KT/HAK/KUP-type potassium transporters, which are associated with fine-tuning of potassium uptake and re-distribution; voltage-independent K^+^ channels of the tandem pore K^+^ (TPK)-type, which are targeted to endomembranes and are assumed to play roles in subcellular osmoregulation and charge equilibration; and finally voltage-gated K^+^ channels of the *shaker*-type that have in common that they are all targeted to the plasma membrane [32]. Detailed information on their expression during seed maturation and on their impact on seed development is not yet ascertained. For this reason, a comprehensive characterization and comparison of all known K^+^ transporters and channels over the course of seed maturation was conducted. Here, we report on the identification of two potassium transporters, HAK/KT12 and KUP4, that contribute to embryonic growth during seed maturation, and the study of their possible regulation by IAA and its precursor, indole-3-acetamide (IAM).

## 2. Results

### 2.1. In Silico-Analysis of K^+^ Transporters Putatively Involved in Seed Maturation

The *A. thaliana* genome contains 13 genes that encode proton-coupled KT/HAK/KUP-transporters, 6 genes coding for voltage-independent TPK-type (tandem pore K^+^) channels, nine genes that encode voltage-gated *shaker*-like K^+^ channels, and one gene coding for a Na^+^/K^+^ transporter of the HKT-type [32]. As a first approach to identify potassium transporting proteins that hold the potential to contribute to an increase in turgor pressure in *Arabidopsis* embryo cells and to be transcriptionally controlled by auxin and/or auxin-related compounds, we performed a two-layered analysis. First, we analyzed expression profiles of the described genes in publicly available microarray repositories [33,34], searching for those genes that show substantial expression during seed development. In particular genes that showed a strong expression during the seed maturation phase (seed stage 8–10: walking-stick to green cotyledons [35]) attracted our attention. Secondly, we searched the databases for information regarding transcriptional responses of the corresponding genes towards IAA treatments (0.5, 1, and 3 hours).

**Figure 1:**
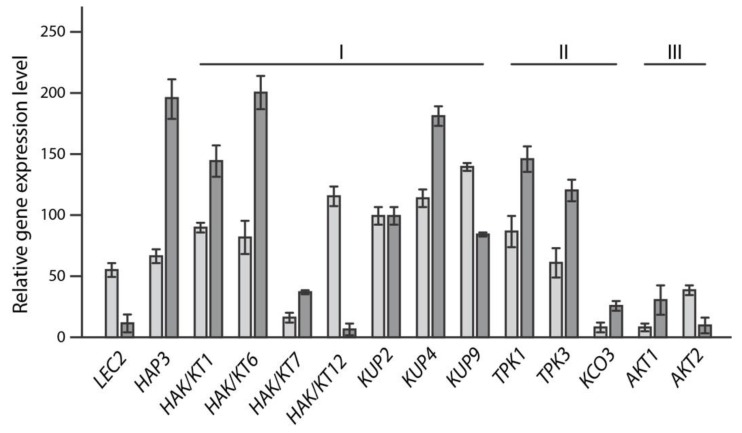
Relative expression of K^+^ transporter/channel genes. Displayed are genes that are expressed during seed development (GEO dataset GSE5634). As references, the expression values of *LEC2* (At1g28300) and *HAP3* (At2g38880) are given. Light grey bars show relative expression levels for siliques with seeds at stage 4 (heart cotyledons), whereas the dark grey bars represent gene transcription levels in seeds without siliques at developmental stage 9. The genes are ordered with respect to their family affiliations. I, KT/HAK/KUP-type transporters; II, voltage-independent tandem pore K^+^ (TPK) channels; III, voltage-gated *shaker*-like K^+^ channels.

Applying the described criteria, it was possible to select a set of 12 genes that showed considerable expression during seed maturation and/or responded to IAA-treatments with changes in their gene expression profiles (Figure 1). Owing to their transcriptional profiles, those genes were selected as candidates that are possibly involved in the rapid cell expansion of embryo cells.

### 2.2. Reverse Genetics Screen to Identify Potassium Transporter/Channel with Significant Impact on Embryo Size

**Figure 2:**
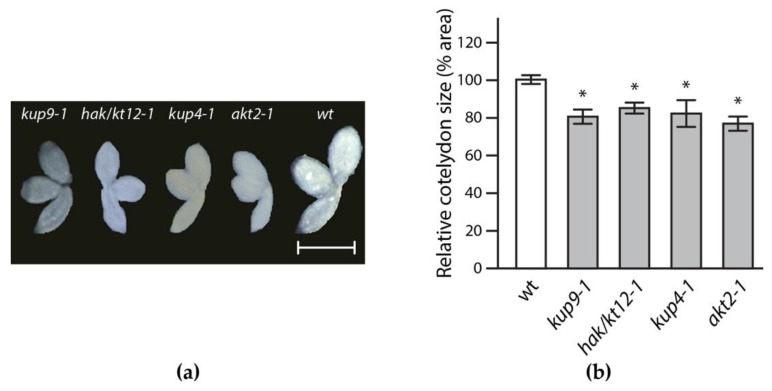
Microscopic analysis of T-DNA insertion mutants. (**a**) Differences in embryo phenotypes of selected T-DNA insertion lines compared to wild-type *Arabidopsis* (wt). Scale bar, 500 µm; (**b**) Quantitative analysis of cotyledon area. Error bars represent the standard error of the mean of three independent experiments, *n* = 12 (Student’s *t*-test; * *p* < 0.05).

To evaluate the influence of the selected transporters/channels on seed maturation and embryo size, two independent *Arabidopsis* T-DNA insertion mutants, either from the Salk Institute knockout (SALK), Syngenta Arabidopsis Insertion Library (SAIL) or Gene Trap collection [36,37,38], for each of the 12 selected target genes were characterized. In most cases, it was possible to isolate and use homozygous mutants in the study (Table 1), underscoring the value of the reverse genetics approach. To analyze the impact of the target gene products, embryos from dry seeds of the mutant lines were inspected. In particular, the cotyledon size was of special interest, since in *Arabidopsis* seeds this is the main organ where storage compounds are deposited. Four out of the 12 investigated targets showed a significant reduction in embryo and cotyledon size, respectively (Figure 2), showing a reduction of the cotyledon area of approximately 20% relative to wild-type *Arabidopsis*. A full overview of the obtained results can be found in Figure S1.

Remarkably, for three of the four identified candidates, *HAK/KT12* (At1g60160), *KUP9* (At4g19960), and *AKT2* (At4g22200), both analyzed T-DNA insertion lines provided similar results. In the case of the *KUP4* gene (At4g23640), only one of the two examined knockout lines showed a significant difference. However, locus effects are the most plausible explanation for this observation. While in the wild type-like mutant (SALK_071644C) the T-DNA was within the first 300 bps upstream of the start codon, the *KUP4* allele that showed an embryo phenotype (SALK_043791C) carried the T-DNA insertion within the first intron. Figure S2 shows the detailed characterization of the eight T-DNA insertion lines for the four identified target genes. The expression of *KUP4* in *kup4-1* (SALK_043791C) was determined to be reduced to approximately 10%, while the *kpu4-2* mutant (SALK_071644C) displayed a remaining *KUP4* expression of 55%. Overall, these results suggest that the identified transporters/channels contribute to embryo cell expansion during seed maturation.

Three of the identified molecular targets, i.e., *HAK*/*KT12*, *KUP4*, and *KUP9*, belong to the KT/KUP/HAK family of proton-coupled potassium transporters [40]. It is assumed that these transporters act in K^+^ uptake and re-distribution, and thus contribute to cellular expansion and/or subcellular targeting [41]. The fourth candidate is *AKT2*, a voltage-gated K^+^ channel of the *Shaker*-type. This channel is known to be able to tap the “potassium battery” in the phloem and energize sugar reloading [42,43]. Moreover, very recently AKT2 has also been associated with long distance electrical signaling in *Arabidopsis* [44], which makes this transporter a particularly interesting object to study.

### 2.3. Identification of Evolutionary Conserved Cis-Motifs in Target Gene Promoters

**Figure 3:**
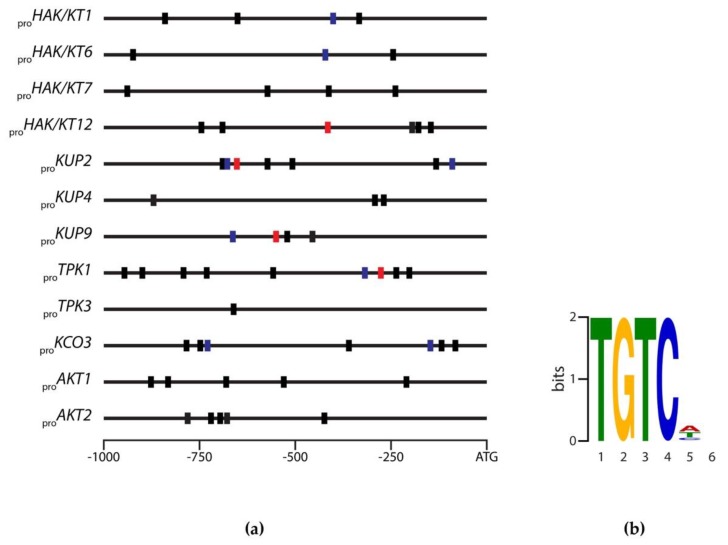
Identification of conserved auxin response factor (ARF) core-binding motifs in target promoters. (**a**) Representation of the 1000 bp promoter fragments upstream of the ATG start codon. Red boxes represent the most frequent TGTCTC consensus sequence, while blue boxes show TGTCGN sequences and black boxes the TGTCNN core sequence motif; (**b**) Position weight matrix (PWM) representation of top-scoring 5-mers for the indicated AuxRE consensus sequence motifs.

Auxin is known to regulate many cellular processes at the transcriptional level [45], including polarization of the zygote during early embryogenesis [46]. Moreover, IAA is assumed to play important roles at later stages of seed development, too. IAA levels have been reported to increase during seed maturation reaching maxima in dry seeds [20]. The *Arabidopsis* genome contains 22 full-length *ARF* genes and one pseudogene (*ARF23*) with a stop codon in its DNA-binding domain. The full-length ARFs share a common domain structure and mediate the primary response to auxin [47,48]. ARFs bind to TGTC-containing *cis*-elements termed auxin response elements (AuxREs) most likely independent of cellular auxin contents [49,50,51]. In order to gain deeper insight into a possible transcriptional control by auxin, the promoter regions of all previously selected target genes were inspected for core AuxRE motifs (Figure 3).

Sequence analysis revealed that the 12 1000 bp-spanning promoter regions contained between one and nine core TGTCNN AuxRE motifs. Among those AuxREs, the *HAK/KT12*, *KUP2*, *KUP9*, and *TPK1* promoters contained the canonical TGTCTC AuxRE [50,52,53]. In addition, the very frequently present TGTCGN consensus sequence was found in the *HAK/KT1*, *HAK/KT6*, *KUP2*, *KUP9*, *TPK1*, and *KCO3* promoters, while no TGTSTSBC motif was detected in the investigated promoters. The TGTSTSBC motif represents an elongated and modified version of the canonical consensus sequence, which has been reported to be overrepresented in about 35% of all auxin responsive genes within the regions 250 bps upstream from the transcriptional start site [49,54]. Notably, the *HAK/KT12*, *KUP2*, *KUP4*, *KUP9*, *TPK1*, *KCO3*, and *AKT2* promoters contained multiple copies of AuxRE motifs in close proximity to each other. The spacing of the motifs in those promoters ranged between 14 and 62 bps. However, they were not organized as either inverted or direct repeats with an spacing of seven to eight nucleotides, which was reported as the optimal spacing for *in vivo* auxin responsiveness [50]. To disclose further context features around the TGTCNN core, the MEME motif discovery algorithm and the GLAM2 algorithm were applied [55,56]. For the identified AuxREs in the 12 studied promoters, 40-nt long sequence fragments with the centrally located TGTCNN hexamer were extracted and analyzed using the MEME Suite 4.12.0 software [57]. Apart from the core TGTCNN motif, the analysis revealed no further positional preferences with respect to the AuxRE motifs detected in the 12 investigated potassium transporter/channel gene promoters.

### 2.4. Transcriptional Regulation of Target Gene Expression during Seed Maturation

**Figure 4:**
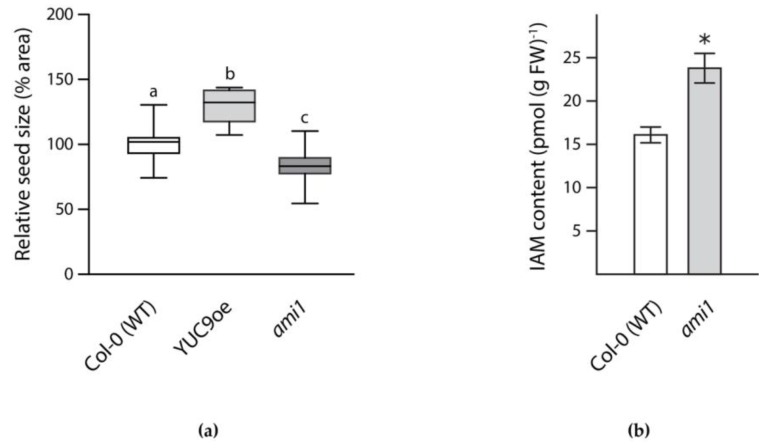
Analysis of auxin mutants. (**a**) Seed size of two selected mutants relative to wild-type *Arabidopsis* seeds. Box plot showing the relative seed size of wild-type (white box), YUC9oe (light grey box), and *ami1* (dark grey box) seeds. The values display the median, quartiles and extremes of the compared data sets, *n* = 16. Different letters indicate significant differences in seed size analyzed by ANOVA and Tukey’s B post-hoc test, *p* < 0.01; (**b**) Quantification of IAM levels in wild-type *Arabidopsis* and the *ami1* T-DNA insertion line. Two-week-old seedlings were used for the determination of IAM by LC-MS analysis. Three independent biological replicates were assessed. Given is the standard error of the mean, *n* = 9 (Student’s *t*-test; * *p* < 0.05).

To underpin our observations regarding the transcriptional regulation of the selected transporter/channel genes by auxin, the response of the 12 previously selected target genes to auxin was analyzed by qRT-PCR. To do so, siliques (seed stage 6–8) were treated with either IAA or IAM. There is mounting evidence that IAM also increases during seed maturation [20] and that artificially increased IAM levels during fruit development promotes parthenocarpy in, e.g., eggplant, tobacco, and tomato [58,59,60,61]. Moreover, an AMI1 homolog protein from *Litchi chinensis* that is suggested to be involved in the conversion of IAM into IAA [62], has recently been associated with lychee parthenocarpy [63]. As shown in Figure 4a, changes in the expression of either *AMI1* or *YUC9* led to significant effects on seed size. While the *ami1* T-DNA insertion mutant [64], which significantly accumulates IAM (Figure 4b), showed a reduced seed size, the *YUC9* overexpression line, which is characterized by a significantly increased endogenous IAA level [65], had seeds that were 20–25% larger. For the given reasons, the possibly negative effect of the IAA precursor, IAM, on potassium transporter/channel gene expression was also examined.

**Figure 5:**
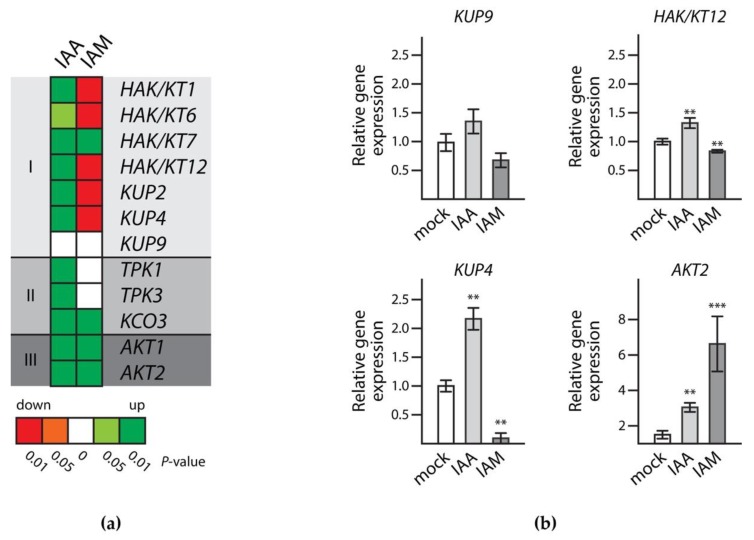
Transcriptional regulation of potassium transporter/channel genes by auxins. (**a**) Relative expression profiles of selected genes in siliques treated with either IAA or IAM. The genes are organized according to their family affiliations. I, KT/KUP/HAK-type transporters; II, tandem pore K^+^ (TPK) channels; III, voltage-gated *Shaker*-like K^+^ channels; (**b**) Exemplary qRT-PCR results for the transcriptional responses of *HAK/KT12*, *KUP4*, *KUP9*, and *AKT2* at 48 h after the treatment with either a mock solution (0.1% EtOH (*v*/*v*)), or 100 µM IAA and IAM, respectively. Data represent relative expression values ± SE, *n* = 9 (Student’s *t*-test; ** *p* < 0.01, *** *p* < 0.001).

First of all, the results indicated that the selected genes were indeed expressed in developing seeds, thereby confirming the microarray data. As shown in Figure 5a, it seemed as if the genes of the different groups of potassium transporters and channels shared common responses to IAA but differed in their response to IAM. While the KT/KUP/HAK family transporter genes were apparently induced by IAA and repressed by IAM, the expression of the tested two pore K^+^ (TPK) channel and *shaker*-type channel genes was induced by IAA, but their response to IAM was either lacking, or the compound also induced their transcription. Taking a closer look at the four candidate genes that showed embryo phenotypes (Figure 5b), it has to be noted that transcriptional changes in *KUP9* expression were not significant. However, in comparison to the two other members of the same group, *KUP9* showed a very similar expression profile in the qRT-PCR assays. The genes *HAK/KT12* and *KUP4* displayed a pronounced induction by IAA of 32% and 117%, while IAM repressed their expression by 17% and 91%, respectively.

On the contrary, the expression of *AKT2* was induced by both compounds, IAA and IAM. Interestingly, the inductive effect of IAM was approximately eight times stronger than the effect triggered by IAA. However, since the expression of *AKT2* appeared to be relatively low in developing seeds, this result should be interpreted with caution. Overall, it can be concluded that the three members of the KT/KUP/HAK transporter family, and here in particular *HAK/KT12* and *KUP4*, were involved in embryo development during seed maturation, and that their expression was most likely controlled by cellular IAA and IAM levels.

**Figure 6:**
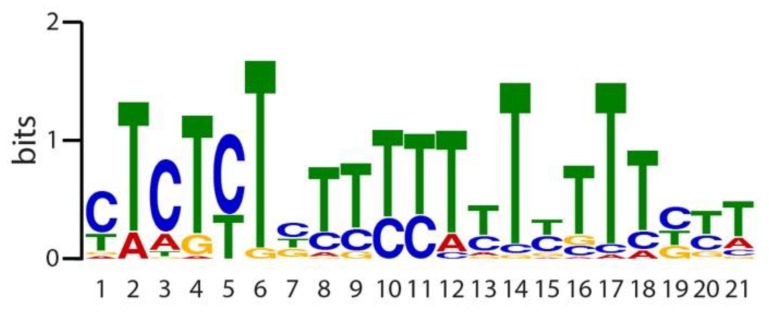
Position weight matrix representation of top-scoring 21-mers for the identified sequence motifs in the promoters of the seven investigated KT/KUP/HAK family transporter genes. The seven 1000 bp spanning promoter regions contain a total of 34 sites of this motif. With an *E*-value of 1.5 × 10^−25^ the motif was the most significant disclosed motif.

The common responses of the investigated KT/KUP/HAK family transporter genes towards IAA and IAM prompted us to search for an additional promoter motif within the 1000 bp promoter fragments upstream of the ATG start codon of the studied transporters of this group that might contribute to responding to IAM. As presented in Figure 6, it was possible to identify an additional, 21 bp comprising motif in all examined promotes.

## 3. Discussion

A total of 29 K^+^ transporters and channels have been identified in the *Arabidopsis* genome [32]. Deeper insight into the function of those transporters/channels is of special interest, since the obtained knowledge might be directly transferable to agriculturally important crops. In this study, we surveyed the 29 genes as potential targets possibly involved in increasing seed size and seed quality. From the initial set of 29 potassium transporters/channels, 12 candidates that are likely implicated in embryo cell expansion were selected based on their developmental expression profiles and responsiveness to IAA. The expression levels of the 17 remaining genes were either very low or non-present in developing seeds (stage 8–10). Due to the recognized rate-limiting role of potassium in crop yield and quality [31], we hypothesized that K^+^ transport is important for proper embryo cell expansion during seed maturation. Hence, the lack or reduction of K^+^ transport is likely to produce visible embryo/seed phenotypes, translating into smaller embryos/seeds. Consequently, two T-DNA insertion lines for each of the selected target genes were characterized, focusing on aberrant embryo/seed phenotypes. From the investigated 24 lines, seven appeared to have a significant seed/embryo phenotype. Remarkably, both examined null-alleles of *HAK/KT12* (SALK_083613C, SALK_045392C), *KUP9* (SALK_108080C, GT_5_94315), and *AKT2* (SALK_017212C, SALK_141384C) showed identical phenotypes, which underlines the reliability of the obtained phenotypic data. For *KUP4*, only one mutant (SALK_043791C) showed a phenotypic difference, while the other line (SALK_071644C) resembled the wild type (Figure S2).

In conclusion, our approach provided evidence for four potassium transporters/channels that affect embryo size during seed maturation in *Arabidopsis*. The quantitative analysis of the T-DNA insertion lines given in Figure S1, revealed an approximately 20% reduced cotyledon area for the different knockout mutants. This observation is particularly relevant as cotyledons function as storage organs in the majority of dicotyledonous plants such as *A. thaliana*. Three of the identified target genes belong to the KT/KUP/HAK transporter family, i.e., *HAK/KT12*, *KUP4*, and *KUP9*. Some members of this transporter family have been associated with important roles in plant development. Because K^+^ is a major cellular solute, impairment in potassium homeostasis may weaken cell turgor and, thus, restrict rates of cell expansion. The role of the KT/KUP/HAK transporters in turgor-dependent growth has been demonstrated in rapidly expanding cotton fibers (*Gossypium hirsutum*) [41]. In agreement with these observations, it is not surprising to find members of this family to be involved in embryo cell expansion, too. KT/KUP/HAK transporters were expected target candidates, owing to those described roles in plant development related to cell expansion growth in other tissues, and their expression profiles in siliques. The expression of *HAK/KT12*, *KUP4*, and *KUP9* during seed maturation could be confirmed by qRT-PCR analysis. In particular, the expression of *HAK/KT12* and *KUP4* was demonstrated to be induced by IAA and repressed in presence of IAM, supporting the notion of IAA:IAM ratios being a regulatory determinant in embryo cell expansion growth. Our observation is backed up by the fact that IAM levels are known to increase during seed maturation, which may mark the end of cell expansion [20]. Interestingly, some other KT/KUP/HAK transporters including *HAK/KT1*, *KUP2*, and *HAK/KT6* show similar responses to IAA and IAM, respectively, in developing siliques (Figure 5a), but do not show obvious embryo phenotypes. Apparently, their role in seed development is most likely only very limited.

In addition, one voltage-gated *shaker*-like K^+^ channel (*AKT2*) turned out to be involved in seed development. *AKT2* acts as a weakly rectifying channel [66,67] and can operate in two different gating modes [68]. In the default mode 1, *AKT2* is a hyperpolarization-activated, inward-rectifying K^+^ channel, which allows the proton pump-driven uptake of potassium. In mode 2, *AKT2* is open and allows the passage of K^+^ ions also out of the cell. The channel can be switched from one mode to the other by a set of post-translational modifications like, e.g., phosphorylation [69,70,71,72]. By switching from gating mode 1 to mode 2 the membrane becomes permeable for K^+^ ions in the entire physiological voltage range, which is equivalent to tapping the transmembrane K^+^ gradient as an additional energy source for transmembrane transport processes [42,43]. With respect to a very recent report, *AKT2* affects also membrane excitability and, together with the outward-rectifying K^+^ release channel GORK, facilitates long-distance electrical signaling [44]. At this point, the other voltage-gated *shaker*-like K^+^ channel gene, *AKT1*, has to be mentioned as well. *AKT1* is a potassium uptake channel [73] and is involved in auxin-mediated root growth inhibition in response to low K^+^ stress [74], which strongly confirms the validity of the here presented transcriptional regulation data. Just like *AKT2*, *AKT1* is considerably expressed during silique development, and the expression of both genes is strongly induced, not only by IAA but also by IAM. Even though only the *akt2* mutants showed an embryo phenotype, both *Shaker*-like K^+^ channels are extremely interesting candidates, due to their extraordinary versatility.

Along with the already mentioned targets, the high expression of *TPK1* and *TPK3* in maturing seeds has to be highlighted (Figure 1). Channels of the TPK/KCO family have been reported to be targeted to the vacuolar membrane [75,76,77]. The physiological role of those channels is still uncertain [78]. Furthermore, another TPK/KCO channel gene, *KCO3*, has to be named, because of its relatively pronounced response to indolic compounds. The function of this channel, however, is currently not fully understood. *KCO3* encodes a truncated TPK protein that lacks essential channel domains. *KCO3* does not appear to have considerable impact on plant growth and development. An isolated *kco3-1* null-allele showed only a weak root growth phenotype, when plants were grown under osmotic stress conditions [79]. Nevertheless, in particular the relation of the target genes with plant stress responses deserves special attention, as IAM has recently also been associated with stress responses of the root system [80].

A particularly interesting outcome of this study was the group-specific differential transcriptional response of the candidate genes to IAA and IAM. While the gene expression of the selected KT/KUP/HAK family transporter genes is largely induced by IAA and repressed by IAM, most of the TPK/KCO family channel genes only responded to IAA, and mostly show no significant response to IAM. On the contrary, the two selected *shaker*-like K^+^ channel genes were induced by both IAA and IAM. The presented results on KT/KUP/HAK transporters might suggest an antagonistic regulatory effect of IAA and IAM in embryo cell expansion that is similar to that of ABA and gibberellic acid (GA) in the control of seed development and germination [81]. This notion of a regulatory effect of IAA and IAM over the course of seed maturation is further supported by a possible crosstalk of IAA with the ABA:GA nexus during seed germination in soybean [82] and a possible control of endogenous IAM level during seed maturation through the transcriptional control of an IAM converting hydrolase (AMI1) by LEC2 [83]. Most striking, however, is the finding that corresponding mutants, which show either elevated endogenous IAM or IAA level, show marked differences in seed size (Figure 4). In order to gain further insight on additional motifs within the promoters of the investigated KT/KUP/HAK family transporters, we conducted a second searched for common motifs in the 1000 bp promoter fragments upstream of the ATG start codon of the investigated transporters. As presented in Figure 6, it was possible to identify an additional motif with a size of 21-bp in all examined promoters. The motif matched with 77 motifs encompassed in the Arabidopsis DAPv1 database [84]. The majority of the identified matches referred to C2C2 and C2H2 zinc finger containing proteins, including a great number of plant-specific DNA binding with one Finger (DOF) transcription factors. It will be highly interesting to examine as to whether higher order mutants of KT/KUP/HAK family transporters show additive effects including stronger embryo phenotypes, and if DOF transcription factors are involved in the IAM-mediated control of gene expression of KT/KUP/HAK family transporters.

On the first sight, the response of the *shaker*-like K^+^ channel genes, which are induced by both IAA and IAM, provides an argument against the IAA:IAM ratio hypothesis. Nevertheless, owing to their membrane voltage-dependent activation and their developmental expression pattern, the functions of *AKT1* and *AKT2* are very difficult to assess and require a more detailed analysis. In particular, the role of *AKT2* needs to be carefully considered in this regard. This is because the null-alleles of *AKT2* show embryo phenotypes (Figures 3a, S1 and S2), and secondly because the expression of *AKT2* is higher at early developmental stages and declines with the onset of seed maturation (Figure 1). While induction by IAM at early developmental stages could play an important role in the establishment and control of the energy household of vascular tissues during embryogenesis [43,85], it may have a subordinate influence at later developmental stages, as *AKT2* expression is relatively low at the end of the seed maturation phase. Work is in progress to obtain more detailed physiological information on the role of *AKT2* in *Arabidopsis* seed development.

## 4. Materials and Methods 

### 4.1. Plant Material and Treatment

All experiments were carried out using *Arabidopsis thaliana* (L.) Heynh. ecotype Col-0 (originally from Nottingham Arabidopsis Stock Centre, NASC, stock N1092) and a number of T-DNA insertion lines provided by the Nottingham Arabidopsis Stock Centre, NASC (cf. Table 1). In addition, a previously published T-DNA insertion line for *AMI1* (*toc64-I*) [64] and a 35S-promoter driver YUC9 overexpression line [65] have been employed. Plants were cultivated in a greenhouse at 22 to 24 °C during daytime and 18 to 20 °C during the night time, with a 16 hours light/8 hours dark cycle. Seeds were germinated in Murashige and Skoog (MS) medium containing 1% (*w*/*v*) sucrose [86]. The photosynthetically active radiation was no less than 150 μE·m^−2^ s^−1^. T-DNA mutants were genotyped as described previously [36]. The expression of the corresponding target genes in the mutant lines was analyzed by qRT-PCR (Figures S2 and S3). Primers for genotyping and expression analysis are given in Table S1.

Treatment of siliques for subsequent qRT-PCR analysis was performed preparing MS solutions of 100 µM IAA and IAM (final EtOH conc. 0.1% (*v*/*v*)) and a mock solution (0.1% EtOH (*v*/*v*)) as a control. Plant material were soaked over 48 h at 4 °C with the corresponding solutions, before the samples were snap-frozen in liquid nitrogen and kept at −80 °C until RNA isolation was executed.

### 4.2. In Silico-Analyses

To assess the expression pattern of the 29 described potassium transporters/channels, corresponding transcript level during seed maturation and IAA responsiveness in young seedlings was studied using public data sources compiling large-scale microarray datasets [33,34]. Expression data were extracted from GEO dataset GSE5634. The 1000 nt stretches proximal to the ATG translational initiation sites of the studied genes were extracted from PlantPromoterDB (PPDB) version 3.0 [87]. Sequences were compared and analyzed using CLC Main Workbench v7.9.2 (QIAGEN). The site recognition tools enoLOGOS [88] and MEME Suite 4.12.0 [57] were applied for the discovery of context pattern around the TGTCNN hexamers in the 18 AuxRE-containing 40-mers extracted from the promoters of the selected genes. To find additional motives in the promoters of the genes of the investigated KT/KUP/HAK family transporters, MEME Suite 4.12.0 was used to disclose context pattern with the first 1000 bps upstream of the start codon. Corresponding motives were subjected to pattern comparison using the Tomtom tool [89], performing a query against an *Arabidopsis* motif database [84].

### 4.3. RNA Isolation and qRT-PCR

Total RNA from siliques was isolated as previously described [90]. The cDNA was synthesized from 2 µg of isolated total RNA by an incubation of 5 min at 70 °C with 1 µL (1 µg) Oligo dT_18_-primer (Promega, Madison, WI, USA) and RNase-free dH_2_O in a total reaction volume of 13.5 µL. Then, 12.5 µL of a mix containing 2.5 mM dNTPs, 40 mM RNAsin, 200 U of Moloney Murine Leukemia Virus (M-MLV) reverse transcriptase and 1 µM of DTT were added. After incubating the solution for 1 h at 37 °C and a subsequent diluted of 1:1 with dH_2_O, samples were incubated for another 5 min at 80 °C and thereafter stored at −20 °C until they were further processed.

Quantitative transcriptional analyses were carried out using complementary DNA (cDNA) synthesized from three independent biological samples, each analyzed in triplicates, on a LightCycler^®^480 (Roche, Basel, Switzerland). Specific forward and reverse primers pairs (20–24 bps) were designed using the QuantPrime tool [91]. *UBIQUITIN 10* (At4g05320) was used as a housekeeping gene for data normalization [92]. Primers and LightCycler^®^480 SYBR Green I Master (Roche) were added to 96 multi-well plates according to the manufacturer’s protocol using 3.5 µL of the samples for a 25 µL reaction volume. Conditions for qRT-PCR were as follows: 10 min at 95 °C, 45 cycles of 15 s at 95 °C, 20 s at 60 °C, and 5 min at 72 °C. A third step for dissociation of 5 s at 95 °C, 1 min at 65 °C, continuous 97 °C, and 30 s at 40 °C was added to analyze melting dynamics.

The threshold cycle (CT) indicates the fractional cycle number at which the amount of amplified target reaches a fixed threshold. That value was used to calculate relative gene expression levels according to the comparative 2^−ΔΔ*C*t^ method [93]. Primers for expression analysis are given in Table S1.

### 4.4. Embryo Preparation and Analysis

Dry seeds from the different T-DNA insertion lines and wild-type *Arabidopsis* were surface sterilized using a 70% ethanol solution (*v*/*v*, 5 min) and a 5–7% sodium hypochloride solution (*v*/*v*, 5 min) and three times rinsed with water. Afterwards they left overnight at 4 °C in DEPC water. Using a stereomicroscope (Leica MZ10 F), seeds were transferred onto an object slide and mixed with glycerol. After removing the testa from the embryo, pictures were taken with 25× magnification using a color closed circuit display (CCD) camera (Leica DFC 420C). Subsequent measurements were made employing the ImageJ image processing software [94].

### 4.5. IAM Quantification

The LC-MS analysis of endogenous IAM was carried out according to the method established by Novak and co-worker [95]. In brief, samples of approx. 100 mg of plant material were harvested and immediately frozen in liquid nitrogen. For each independent experiment three biological replicates were analyzed. The plant material was transferred into 2 mL screw-cap tubes filled with ceramic beads (MagNA Lyser Green Beads) and IAM was extracted into 1 mL of cold potassium phosphate buffer (50 mM, pH 7.0) containing 1% diethyldithiocarbamic acid sodium salt, and 50 pmol of [^2^H_5_,^15^N]-IAM (OlchemIm, Olomouc, Czech Republic) as internal standard. The plant material was crushed in a MagNA Lyser Instrument (Roche) at 6,500 rpm for 1 min. After centrifugation (15 min, 16,000× *g* at 4 °C), 750 µL of the supernatants were transferred into fresh tubes and the pH adjusted to 2.7 with 1 M hydrochloric acid. The samples were loaded onto Oasis^TM^ HLB columns (1 mL, 30 mg, Waters, Milford, MA, USA) conditioned with 1 mL methanol and 1 mL water, and equilibrated with 1 mL acidified sodium phosphate buffer (50 mM, pH 2.7). The columns were washed twice with 1.5 mL 5% methanol and subsequently eluted with 2 mL 80% methanol. The eluates were taken to dryness *in vacuo*. Prior to the mass spectrometric analysis, the evaporated samples were dissolved in 45 µL methanol with 0.1% formic acid (*v*/*v*). Ten microliters of the extract were separated using an Ultimate3000 RSLC system (Dionex, Sunnyvale, CA, USA). The column used was a 50 mm × 2.1 mm i.d., 1.7 μm, Acquity UPLC BEH C18 column with a 5 mm × 2.1 mm i.d. Acquity UPLC BEH C18 VanGuard pre-column, and a 0.2 μm × 2.1 mm i.d. in-line filter (Waters). The following binary gradient was applied: 0 to 2 min isocratic 98% solvent A (water with 0.1% (*v*/*v*) formic acid), 2% B (acetonitrile with 0.1% (*v*/*v*) formic acid); 2 to 30 min linear gradient to 5% A, 95% B; at 30 min step gradient to 100% B; isocratic for 1 min. Thereafter, the column was set to 98% A, 2% B and conditioned for 2 min before the next injection. The flow rate was 400 μL min^−1^. Eluted compounds were analyzed by a microTOF-Q II mass spectrometer (Bruker Daltonics, Bremen, Germany) operated in positive electrospray mode. Typical settings were as follows: capillary voltage, −4,500 V; dry gas temperature, 200 °C; dry gas flow, 10 L min^-1^; funnel, RF 200 Vpp. Ions were detected from *m/z* 50 to 1000 at a repetition rate of 2 Hz. The instrument was operated in MRM mode. Mass calibration was performed using sodium formate clusters (10 mM solution of NaOH in 50/50% *v*/*v* isopropanol/water containing 0.2% formic acid). For data processing the DataAnalysis 4.0 software (Bruker Daltonics) was used. The following transitions were recorded to determine analyte contents: IAM, *m/z* = 175.2 → 130.1 [^2^H_5_,^15^N]-IAM, *m*/*z* = 181.2 → 135.1 (Retention time, 6.3 min). Quantification was achieved by comparing peak areas of the endogenous compounds with those of the internal standards.

### 4.6. Statistical Analysis

The obtained data were analyzed using univariate statistics by employing either Student’s *t*-test to allow for comparison of two means, or ANOVA followed by Tukey’s B post-hoc test to allow for comparisons among all means. Statistical analysis was conducted using PRISM v5.03 (GraphPad Software, http://www.graphpad.com/). Significance thresholds are indicated in the figure legends.

## Figures and Tables

**Table 1:** Analyzed transfer DNA (T-DNA) insertion mutants.

NASC Code	Name	Genotype	Gene
N662453	*hak/kt1-1*	SALK_051343C/homozygous	*HAK/KT1* At2g30070
N648762	*hak/kt1-2*	SALK_148762/heterozygous	*HAK/KT1* At2g30070
N561656	*hak/kt6-1*	SALK_061656/homozygous	*HAK/KT6* At1g70300
N586950	*hak/kt6-2*	SALK_086950/homozygous	*HAK/KT6* At1g70300
N805085	*hak/kt7-1*	SAIL_105_G04/homozygous	*HAK/KT7* At5g09400
N671076	*hak/kt7-2*	SALK_004133C/homozygous	*HAK/KT7* At5g09400
N656697	*hak/kt12-1*	SALK_083613C/homozygous	*HAK/KT12* At1g60160
N665909	*hak/kt12-2*	SALK_045392C/homozygous	*HAK/KT12* At1g60160
N670400	*kup2-1*	SALK_023287C/homozygous	*KUP2* At2g40540
N597636	*kup2-2*	SALK_097636/heterozygous	*KUP2* At2g40540
N670640	*kup4-1*	SALK_043791C/homozygous	*KUP4* At4g23640
N684136	*kup4-2*	SALK_071644C/homozygous	*KUP4* At4g23640
N670022	*kup9-1*	SALK_108080C/homozygous	*KUP9* At4g19960
N163575	*kup9-2*	GT_5_94315 [38]	*KUP9* At4g19960
N9729	*tpk1-1*	SALK_146903/homozygous	*TPK1* At5g55630
N661151	*tpk1-2*	SALK_131790C/homozygous	*TPK1* At5g55630
N662409	*tpk3-1*	SALK_049137C/homozygous	*TPK3* At4g18160
N663176	*tpk3-2*	SALK_085696C/homozygous	*TPK3* At4g18160
N684833	*kco3-1*	SALK_048607C/homozygous	*KCO3* At5g46360
N596038	*kco3-2*	SALK_096038/heterozygous	*KCO3* At5g46360
N3762	*akt1-1*	*akt1-1* [39]/homozygous	*AKT1* At2g26650
N686273	*akt1-2*	SALK_071803C/homozygous	*AKT1* At2g26650
N673953	*akt2-1*	SALK_017212C/homozygous	*AKT2* At4g22200
N679170	*akt2-2*	SALK_141384C/homozygous	*AKT2* At4g22200

## Supplementary Materials

Supplementary materials can be found at http://www.mdpi.com/1422-0067/19/7/2132/s1.

## Abbreviations

cDNA	complementary DNA
EtOH	ethanol
DTT	1,4-dithiothreitol
DEPC	diethyl pyrocarbonate
qRT-PCR	quantitative reverse transcriptase polymerase chain reaction
T-DNA	transfer DNA
