# Supplementary figures and images for: Identification of Two Auxin-Regulated Potassium Transporters Involved in Seed Maturation
Source: Int J Mol Sci. 2018 Jul 22;19(7):2132. doi: 10.3390/ijms19072132 (PMC6073294; doi:10.3390/ijms19072132)

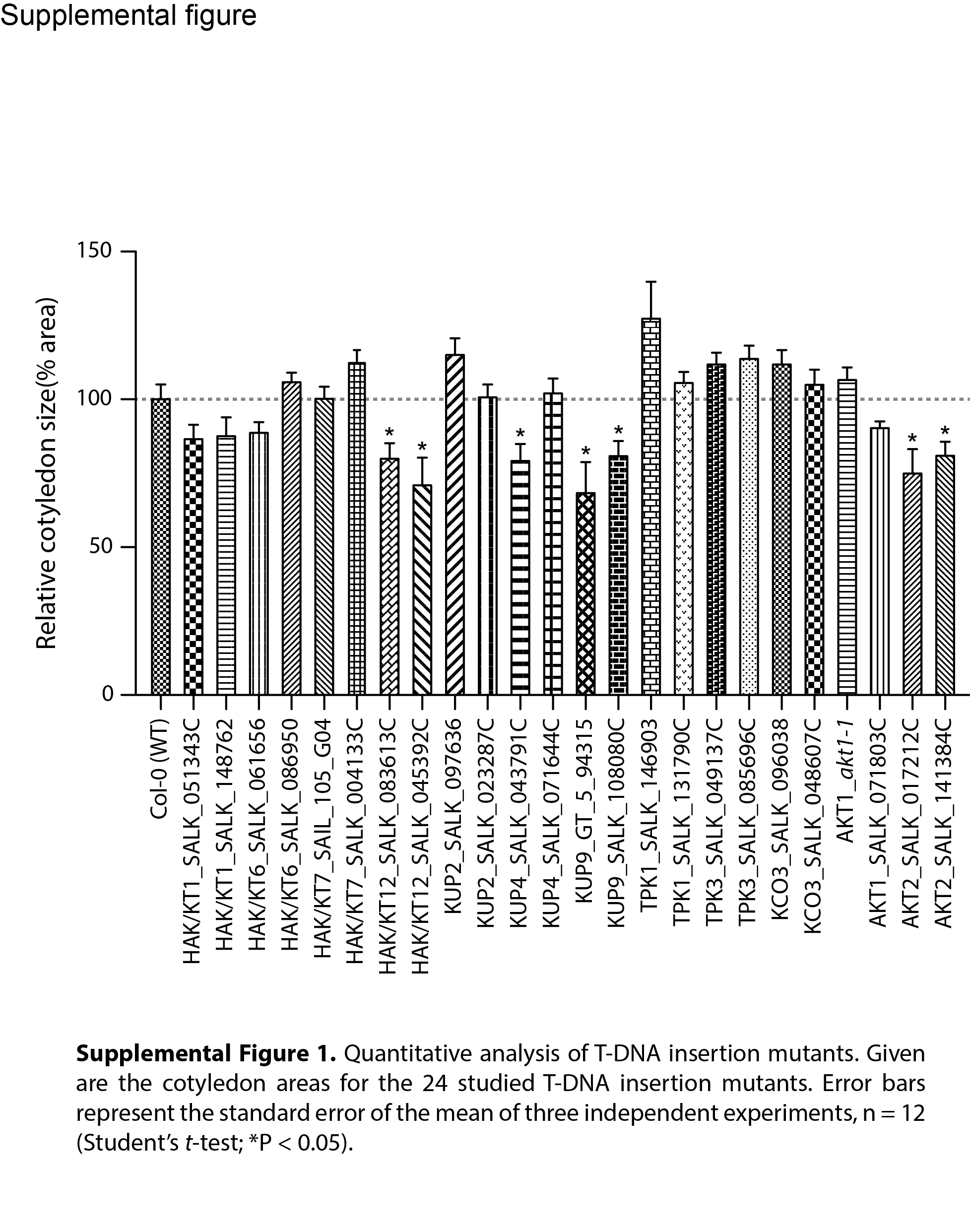


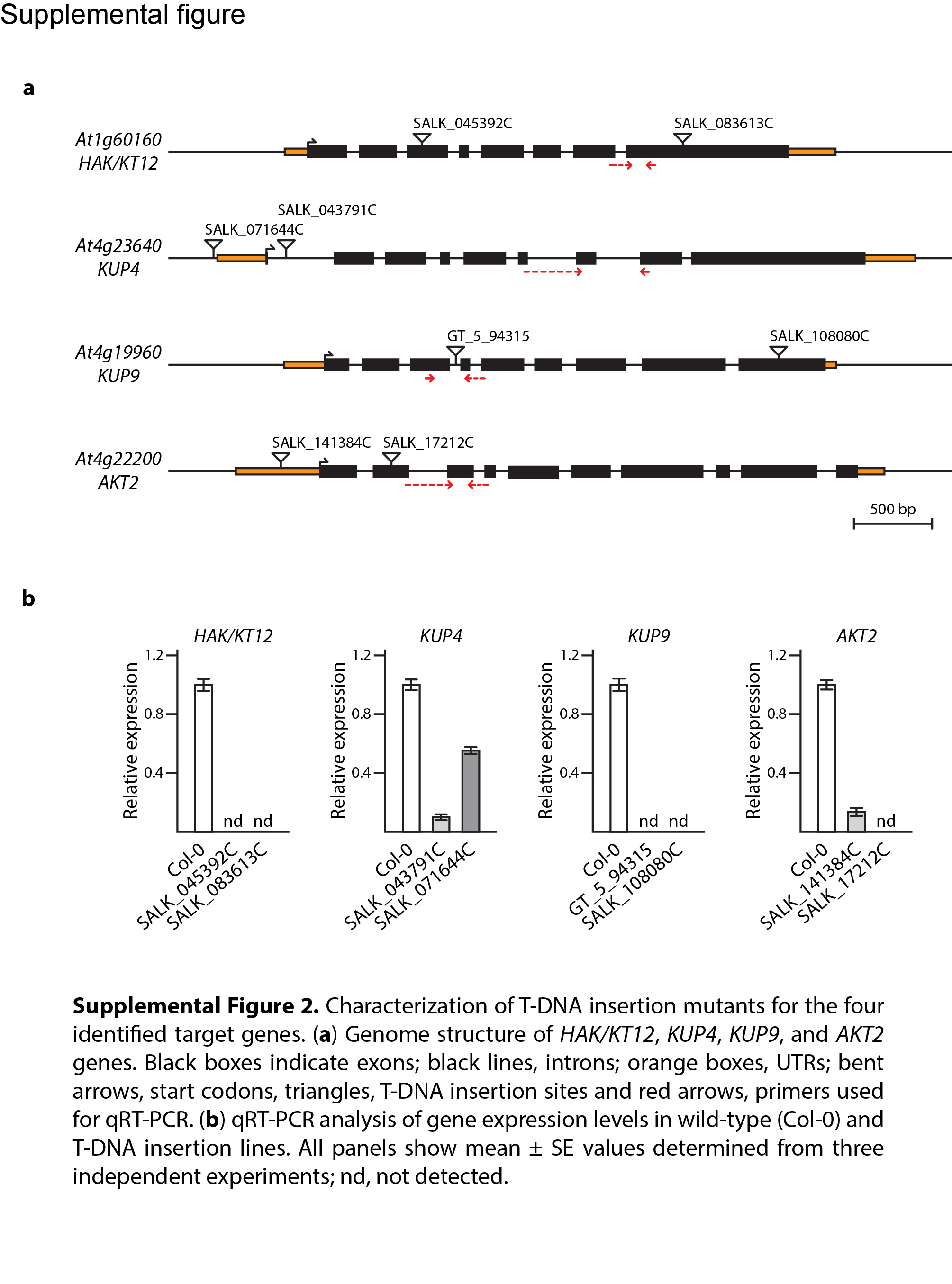


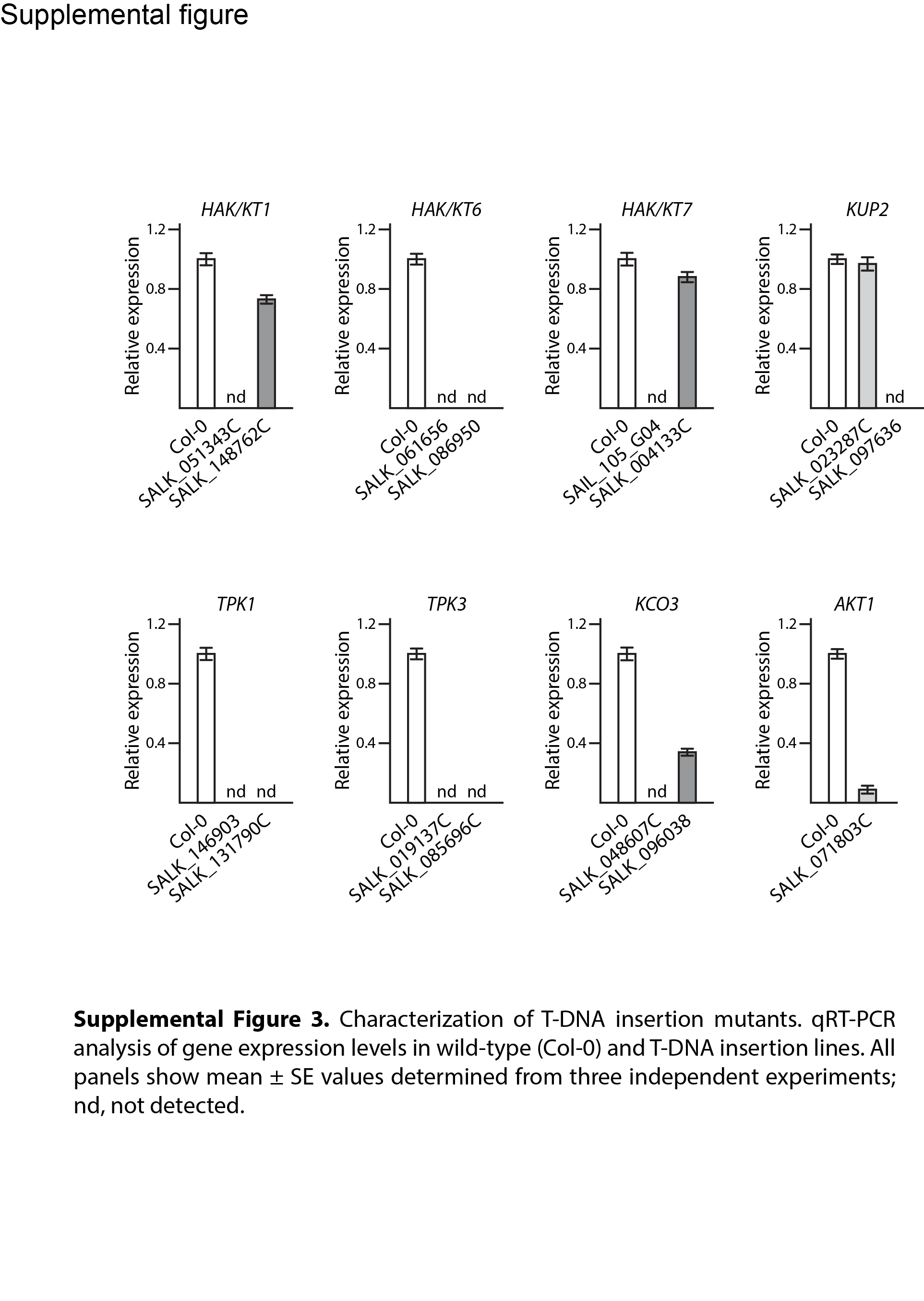

Supplement: Supplementary file 1 [file ijms-19-02132-s001.zip › Supplemental figures.docx]
